# Assessing Alexithymia: Psychometric Properties of the Perth Alexithymia Questionnaire in a Spanish-Speaking Sample

**DOI:** 10.3389/fpsyt.2021.710398

**Published:** 2021-10-12

**Authors:** Rodrigo Becerra, Carmen Gloria Baeza, Ana Maria Fernandez, David A. Preece

**Affiliations:** ^1^School of Psychological Science, University of Western Australia, Perth, WA, Australia; ^2^School of Psychology, University of Santiago, Santiago, Chile; ^3^School of Psychology, Curtin University, Perth, WA, Australia

**Keywords:** alexithymia, Perth alexithymia questionnaire, Spanish, factor structure, emotion

## Abstract

Alexithymia is a trait composed of difficulties identifying feelings (DIF), difficulties describing feelings (DDF), and externally orientated thinking (EOT). It is an important transdiagnostic risk factor for psychosomatic disorders and other types of emotion-based psychopathologies, and can reduce the efficacy of some treatment approaches. Alexithymia assessments are therefore important in psychiatric and research settings. The Perth Alexithymia Questionnaire (PAQ) was recently developed to enable more comprehensive alexithymia assessments, however, its psychometric properties need further examination and it is so far only available in English. In this study, we sought to address this by translating the PAQ into Spanish and testing its psychometric properties in an adult sample from Chile (*N* = 370). Confirmatory factor analyses found the PAQ to have a theoretically congruent factor structure, supporting the contemporary status of alexithymia as a multifaceted construct and the PAQ's capacity to assess the DIF, DDF, and EOT facets of alexithymia across negative and positive emotions. All subscale and composite scores had high internal consistency reliability, and demonstrated good concurrent and discriminant validity. The PAQ therefore appears to provide a robust and detailed alexithymia profile. This Spanish version should help to enable more comprehensive cross-cultural research into alexithymia and its role in and psychological disorders.

## Introduction

Alexithymia is a trait composed of three facets: difficulty identifying one's own feelings (DIF), difficulty describing feelings (DDF), and an externally orientated thinking style (EOT) whereby one rarely focuses attention on their internal emotional states (1–4). People with high alexithymia experience their emotions in a more diffuse or undifferentiated manner, such as being unsure if an unpleasant feeling is sadness, anger, or fear (5). Alexithymia was first coined by American psychiatrists in the 1970s, who often observed high levels of the trait in treatment resistant patients with psychosomatic disorders (4, 6). The relevance of the valid assessment of alexithymia in psychiatry in general and psychosomatic disorders in particular, becomes apparent upon inspection of the prevalence of alexithymia in these domains. Alexithymia has been found to be overrepresented in numerous psychiatric disorders, including substance abuse (7), alcohol dependence (8), depression (9), anxiety disorders (10), addictive disorders in general (11), and in general psychiatric samples with varied diagnoses (12). Similarly, alexithymia has been found to be present in numerous medical presentations with associations with psychosomatic conditions, including gastrointestinal problems (13), dermatological issues (14), and cardiovascular complications (15). Importantly, the presence of high alexithymia has been found to impair treatment effectiveness if unaccounted for [e.g., (16)]. Therefore, the role of alexithymia in clinical practice appears to warrant its close examination from both the intervention and assessment points of view. Assessing alexithymia should thus be relevant to clinicians working with psychiatric patients and/or patients with medical illnesses found to have associations with psychosomatic conditions.

Since the 1990s, researchers and practitioners have most frequently used the 20-item Toronto Alexithymia Scale [TAS-20; (17)] to assess alexithymia, a strength of which is its availability in 24 languages. However, researchers and practitioners are increasingly wanting to examine alexithymia at the facet (subscale) level as well as conduct valence-specific analyses [i.e., examine DIF, DDF, and EOT across both negative and positive emotions; (18–20)]. This parallels trends in the broader psychological assessment field, where contemporary assessment tools are increasingly providing valence-specific scores and emphasizing facet level profiles for other multidimensional emotional constructs [e.g., emotion regulation, emotional reactivity, beliefs about emotions; (21–25)]. However, the TAS-20 was not designed with these functions in mind [it was designed only to provide a total scale score as a measure of overall alexithymia; see (26)] and psychometric studies have consistently noted low reliability (α < 0.70) and factor loading problems with its EOT items [e.g., (27–33)]. Furthermore, recent data have suggested that a significant portion of the variance in the TAS-20 DIF items reflects people's current levels of negative affect rather than their underlying levels of the alexithymia trait [i.e., discriminant validity problems; see (34–36, 50)].

To try to enable more detailed facet-level and valence-specific analyses of alexithymia, Preece et al. (37) recently developed the Perth Alexithymia Questionnaire (PAQ). The PAQ is a 24-item self-report measure designed to assess all three components of the construct (DIF, DDF, EOT). For the DIF and DDF components, valence-specific scores can be derived for processing negative and positive emotions. Thus, the PAQ has five intended subscales: *Negative-Difficulty identifying feelings* (N-DIF; 4 items, e.g., “When I'm feeling bad, I can't tell whether I'm sad, angry, or scared”), *Positive-Difficulty identifying feelings* (P-DIF; 4 items, e.g., “When I'm feeling good, I can't make sense of those feelings”), *Negative-Difficulty describing feelings* (N-DDF; 4 items, e.g., “When I'm feeling bad, if I try to describe how I'm feeling I don't know what to say”), *Positive-Difficulty describing feelings* (P-DDF; 4 items, e.g., “When I'm feeling good, I can't talk about those feelings in much depth or detail”), and *General-Externally orientated thinking* (G-EOT; 8 items, e.g., “I prefer to focus on things I can actually see or touch, rather than my emotions”). These subscales can also be combined into several composite scores, including summing all items into a total scale score as an overall marker of alexithymia. All PAQ items are answered on a 7-point Likert scale, with higher scores indicating higher levels of alexithymia. The scale takes approximately between 3 and 5 min to complete, therefore it can be used in a non-intrusive manner in any clinical setting.

The psychometric properties of the PAQ have so far been examined in four published studies (34, 37–39), all using the original English version with Australian or United States adults. The PAQ has displayed a theoretically congruent structure, consisting of five narrow factors (corresponding to the five intended subscales), with support also found for a bifactor model with an additional general alexithymia factor loading on all items (37)^1^. All subscale and composite scores in these studies had high internal consistency, and good concurrent and discriminant validity against measures of depression and anxiety symptoms and emotion regulation. Supporting the potential value of valence-specific assessment, participants reported more difficulties identifying and describing their negative emotions as compared to positive emotions, and the PAQ displayed better reliability coefficients and factorial validity than the TAS-20 (34, 38, 39).

Whilst these results are promising, there is a need for further validation studies to confirm the PAQ's utility. The PAQ is presently also only available in English, which limits cross-cultural applications. To help address these gaps, in this study we translated the PAQ into Spanish and examined its psychometric properties in a Spanish-speaking sample. Options for alexithymia assessments in Spanish-speaking populations are presently limited [e.g., a recent study of the Spanish TAS-20 found the aforementioned factorial validity and reliability problems, with the authors recommending that the scale “needs improvement (theoretical and empirical) to ensure optimal indices”; (28), p.1; see also (42)]. Thus, there is a pressing need to develop more optimized measures for alexithymia assessments in Spanish-speaking populations.

## Method

### Participants, Procedure, and Materials

Ethics approval for this study was granted by the Human Research Ethics Committee from the University of Santiago. All participants provided informed consent. The first author, who is a Spanish-English bilingual psychologist with expertise in scale development, translated the English PAQ items into Spanish. This Spanish translation was then sent to the second and third authors (both Spanish-English bilingual psychologists) who suggested minor modifications. This version was back-translated into English, and checked by the first author and last author (an English-speaking psychologist with expertise in scale development). The final version of the Spanish PAQ (see Supplementary Materials) was administered to 370 Spanish speaking adults (63.2% female) in Chile. Their average age was 28.14 years (*SD* = 11.97, range = 18–66). The sample was 55% undergraduate students (from the School of Humanities at the University of Santiago), 25% professionals, and 20% technical workers or homemakers. 79% of the sample were single, 16% married, and 5% divorced or separated. Less than 6% of the sample reported having a psychological disorder, which included depression (*n* = 11), anxiety (*n* = 4), both anxiety and depression (*n* = 2), bipolar disorder (*n* = 2), ADHD (*n* = 1), and Asperger syndrome (*n* = 1).

As a concurrent and discriminant validity marker, participants also completed a measure of emotional reactivity, the Perth Emotional Reactivity Scale [PERS; (43)]^2^. The PERS is a 30-item self-report questionnaire that assesses the typical *ease of activation, intensity*, and *duration* of people's emotions, and does so for negative and positive emotions separately. Higher scores indicate higher levels of emotional reactivity. The PERS has demonstrated good validity and reliability [e.g., (43)].

### Analytic Strategy

Confirmatory factor analyses (CFA) were conducted using AMOS 25. All other analyses used SPSS 25.

#### Factorial Validity

**Figure 1:**
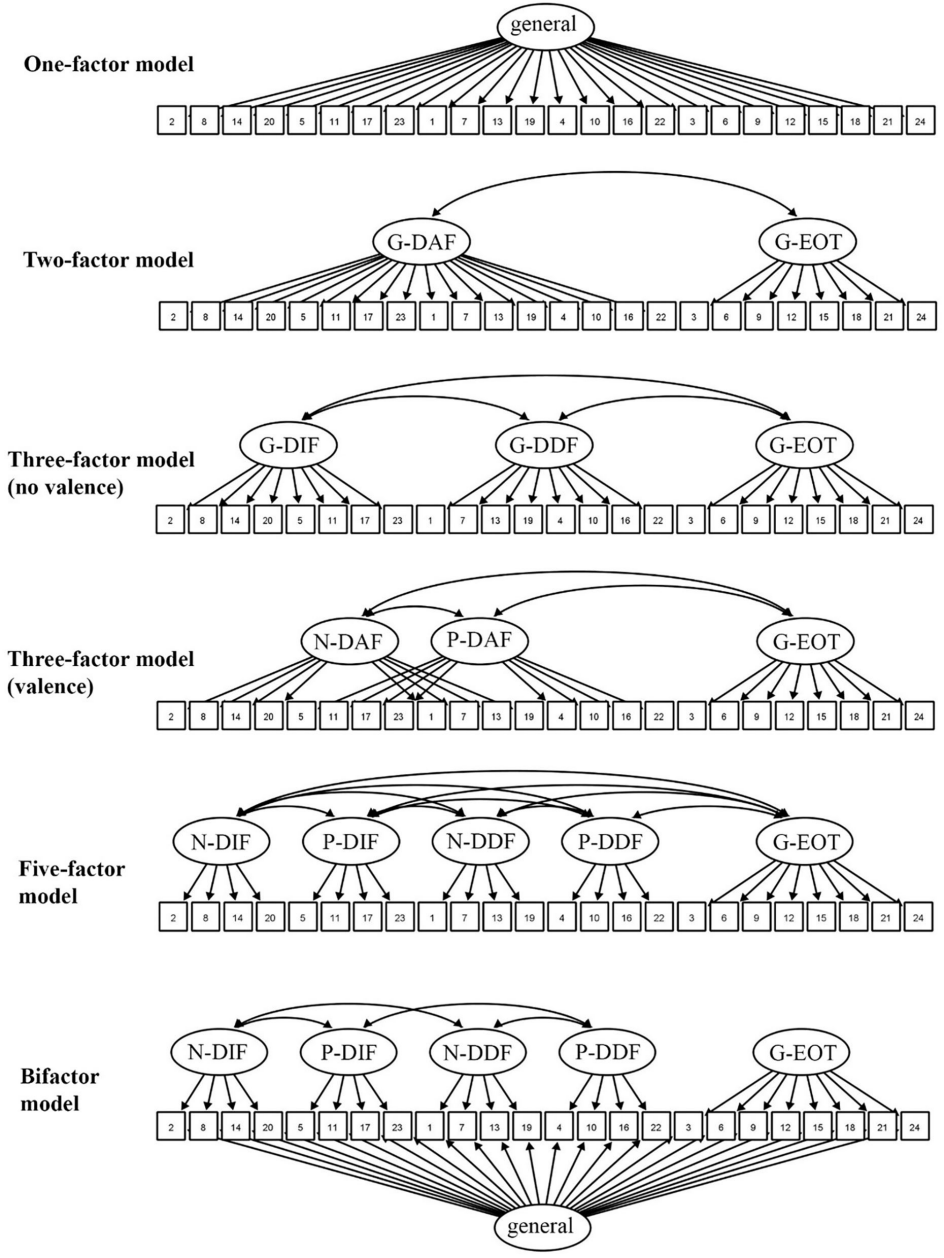
The examined confirmatory factor analysis models for the Perth Alexithymia Questionnaire. Ellipses = latent factors, squares = item numbers. Each item had an associated error term.

We examined the factor structure of the PAQ using a series of CFAs (maximum likelihood estimation based on a Pearson covariance matrix), following the same statistical and model testing procedure as Preece et al.'s (37) original PAQ development study^3^. We examined six theoretically informed models of increasing complexity (see Figure 1). As comparative baselines, first we tested several simpler models: a *one-factor model*, where all items loaded on single factor; a *two-factor model*, that only distinguished between the attention (EOT) and appraisal (DIF, DDF) stages of emotion processing; a *three-factor non-valenced model*, that distinguished between the DIF, DDF and EOT facets of alexithymia, but did not distinguish between negatively and positively valenced items; and a *three-factor valenced model*, that combined the DIF and DDF items together, but distinguished between the processing of negative (N-DIF/N-DDF) and positive emotions (P-DIF/P-DDF). Then, we tested two models reflecting the intended factor structure of the PAQ: a *five-factor model*, comprised of the five intended subscales as correlated factors (N-DIF, P-DIF, N-DDF, P-DDF, G-EOT), and a *bifactor* version of this model, where a general factor was also included loading on all the items. The bifactor model was the best solution in Preece et al.'s (37) original development study, so we expected it to be the best here.

The goodness-of-fit of each model was judged based on four fit index values: CFI, TLI, RMSEA, and SRMR. CFI and TLI values around ≥0.90 indicate acceptable fit. RMSEA and SRMR values around ≤ 0.08 are acceptable. AIC values were also used to directly compare each model, with lower AIC values indicating a better fit (44, 46). Factor loadings ≥0.40 were judged as meaningful loadings (47).

#### Internal Consistency

Cronbach's α reliability coefficients were calculated for all PAQ subscale and composite scores. Coefficients ≥0.70 were viewed as acceptable, ≥0.80 as good, and ≥0.90 as excellent (48).

#### Concurrent and Discriminant Validity

We calculated Pearson correlations between PAQ and PERS scores. People's emotion regulation attempts are typically focused on up-regulating negative emotions and down-regulating positive emotions, and because alexithymia impairs emotion regulation abilities people with high alexithymia should be less successful at achieving these regulation goals (49). Alexithymia should therefore be associated with an emotional reactivity profile characterized by high negative reactivity (i.e., more easily activated, more intense, and more prolonged negative emotions) and low positive reactivity (49). Alexithymia measures should, however, still assess a construct that is separable from emotional reactivity [i.e., should demonstrate discriminant validity in terms of not being a measure of negative or positive affect; (50)]. We examined this discriminant validity by conducting a second-order exploratory factor analysis (EFA; principal axis factoring with direct oblimin rotation) of the PAQ and PERS subscale scores together, to see whether their subscales successfully extracted onto different factors.

## Results and Discussion

### Factorial Validity

Our CFA results replicated the previous findings of Preece et al. with the English PAQ. Those models which included the five intended subscales as factors (i.e., the five-factor model and the bifactor model) were the best solutions. Fit index values, factor loadings, and factor intercorrelations are provided in Tables 1, 2, and Supplementary Table 1, respectively. The simpler models that did not account for valence had poor fit (e.g., CFI <0.90). In contrast, the five-factor model fit well according to all examined fit indexes [χ^2^ = 702.485 (*p* < 0.001), CFI = 0.921, TLI = 0.910, RMSEA = 0.072 (0.066–0.078), SRMR = 0.0483, AIC = 818.785], thus highlighting that it was useful to distinguish between the DIF, DDF and EOT components of alexithymia, and distinguish between the processing of negative and positive emotions for DIF and DDF. All items in the five-factor model loaded well on their intended factor (factor loadings = 0.60–0.89) and these five factors were positively correlated (estimated *rs* = 0.47–0.91). The addition of the general factor in the bifactor model improved model fit further [χ^2^ = 636.515 (*p* < 0.001), CFI = 0.929, TLI = 0.913, RMSEA = 0.071 (0.064–0.077), SRMR = 0.0522, AIC = 788.515], and item variance was split well between the five narrow factors and the general factor (see Table 2). Overall, our CFA results were therefore consistent with the theoretical status of alexithymia as a coherent multidimensional construct (1, 4, 41), and supported the intended subscale and composite score structure of the PAQ. Taken together with previous factor analytic findings in English-speaking samples, our results therefore support the consistency of the structure of alexithymia across these cultural groups.

**Table 1:** Goodness-of-fit index values for the different confirmatory factor analysis models of the Perth alexithymia questionnaire.

**Model**	**χ^**2**^ (*df*)**	**CFI**	**TLI**	**RMSEA**	**SRMR**	**AIC**
One-factor model	238.294 (252)	0.643	0.609	0.150 (0.144–0.155)	0.1089	2434.294
Two-factor model	1,672.182 (251)	0.757	0.733	0.124 (0.118–0.130)	0.0917	1770.182
Three-factor non-valenced model	1,639.068 (249)	0.762	0.737	0.123 (0.117–0.129)	0.0932	1741.068
Three-factor valenced model	820.042 (249)	0.902	0.892	0.079 (0.073–0.085)	0.0502	922.042
Five-factor model	702.785 (242)	0.921	0.910	0.072 (0.066–0.078)	0.0483	818.785
Bifactor model	636.515 (224)	0.929	0.913	0.071 (0.064–0.077)	0.0522	788.515

**Table 2:** Standardized factor loadings from confirmatory factor analyses of the Perth alexithymia questionnaire (five-factor model and bifactor model).

**Factor/item**	**Five-factor model**	**Bifactor model**
**Negative-difficulty identifying feelings**		
2-When I'm feeling *bad*, I can't tell whether I'm sad, angry, or scared. *Cuando me siento mal, no se si estoy trizte, enojado, o asustado*.	0.61	0.51 (0.35)
8-When I'm feeling *bad*, I can't make sense of those feelings. *Cuando me siento mal, no logro entender esas emociones*.	0.81	0.58 (0.57)
14- When I'm feeling *bad*, I get confused about what emotion it is. *Cuando me siento mal, me confundo acerca de que emocion estoy sintiendo*	0.81	0.68 (0.46)
20-When I'm feeling *bad*, I'm puzzled by those feelings. *Cuando me siento mal, quedo perplejo con esas emociones*	0.85	0.68 (0.52)
**Positive-difficulty identifying feelings**		
5-When I'm feeling *good*, I can't tell whether I'm happy, excited, or amused. *Cuando me siento bien, no se si estoy feliz, emocionado, o divertido*.	0.60	0.46 (0.38)
11-When I'm feeling *good*, I can't make sense of those feelings. *Cuando me siento bien, no logro entender esas emociones*.	0.78	0.55 (0.55)
17- When I'm feeling *good*, I get confused about what emotion it is. *Cuando me siento bien, me confundo acerca de que emocion estoy sintiendo*	0.79	0.61 (0.52)
23- When I'm feeling *good*, I'm puzzled by those feelings. *Cuando me siento bien, quedo perplejo con esas emociones*	0.84	0.63 (0.56)
**Negative-difficulty describing feelings**		
1-When I'm feeling *bad* (feeling an unpleasant emotion), I can't find the right words to describe those feelings. *Cuando me siento mal (con una emocion desgradable), no puedo encontrar las palabras adecuadas para describir esas emociones*.	0.66	0.49 (0.43)
7-When I'm feeling *bad*, I can't talk about those feelings in much depth or detail. *Cuando me siento mal, no puedo hablar acerca de esos sentimientos con profundidad o detalle*.	0.77	0.56 (0.52)
13-When something *bad* happens, it's hard for me to put into words how I'm feeling. *Cuando algo malo ocurre, me cuesta encontrar las palabras para describir como me estoy sintiendo*	0.84	0.59 (0.59)
19-When I'm feeling *bad*, if I try to describe how I'm feeling I don't know what to say. *Cuando me siento mal, si trato de describir lo que siento, no se como decirlo*	0.89	0.68 (0.58)
**Positive-difficulty describing feelings**		
4- When I'm feeling *good* (feeling a pleasant emotion), I can't find the right words to describe those feelings. *Cuando me siento bien (con una emocion agradable), no puedo encontrar las palabras adecuadas para describir esas emociones*.	0.67	0.50 (0.44)
10- When I'm feeling *good*, I can't talk about those feelings in much depth or detail. *Cuando me siento bien, no puedo hablar acerca de esos sentimientos con profundidad o detalle*.	0.71	0.50 (0.51)
16- When something *good* happens, it's hard for me to put into words how I'm feeling. *Cuando algo bueno ocurre, me cuesta encontrar las palabras para describir como me estoy sintiendo*	0.82	0.56 (0.60)
22- When I'm feeling *good*, if I try to describe how I'm feeling I don't know what to say. *Cuando me siento bien, si trato de describir lo que siento, no se como decirlo*	0.83	0.60 (0.58)
**General-externally orientated thinking**		
3-I tend to ignore how I feel. Tiendo a ignorar como me siento	0.70	0.34 (0.61)
6-I prefer to just let my feelings happen in the background, rather than focus on them. *Prefiero dejar que mis sentimientos ocurran en el fondo, en vez de prestarles atencion*.	0.71	0.53 (0.55)
9-I don't pay attention to my emotions. *No le presto atencion a mis emociones*.	0.84	0.61 (0.68)
12-Usually, I try to avoid thinking about what I'm feeling. *Generalmente, trato de no pensar acerca de lo que estoy sintiendo*.	0.75	0.31 (0.68)
15-I prefer to focus on things I can actually see or touch, rather than my emotions. *Prefiero prestar atencion a cosas que puedo puedo ver o tocar, en vez de mis emociones*.	0.77	0.18 (0.75)
18-I don't try to be “in touch” with my emotions. *Trato de no estar en contacto con mis emociones*.	0.73	0.15 (0.73)
21-It's not important for me to know what I'm feeling. *No es importante para mi saber que estoy sintiendo*.	0.76	0.18 (0.75)
24-It's strange for me to think about my emotions. *Es extrano para mi pensar acerca de mis emociones*.	0.84	0.24 (0.81)

*All factor loadings were statistically significant, p < 0.05. For the bifactor model, item factor loadings outside of the brackets are the loadings on the five narrow subscale factors, and item factor loadings inside the brackets are the loadings on the general factor*.

### Descriptive Statistics, Reliability, and Concurrent/Discriminant Validity

Descriptive statistics and Cronbach's α coefficients are provided in Table 3. All PAQ subscales and composite scores had good to excellent levels of reliability (α = 0.83–0.94). Consistent with previous findings (37–39), participants reported significantly more difficulties appraising their negative emotions compared to their positive emotions [*t*_(369)_ = 9.950, *p* < 0.001, *d* = 0.52], thus reinforcing the utility of valence-specific measurement.

**Table 3:** Descriptive statistics and Cronbach's α reliability coefficients for the Perth alexithymia questionnaire.

**Subscale/composite**	**No. of items**	***M***	***SD***	**Range**	**α**
**Subscales**					
Negative-Difficulty identifying feelings (N-DIF)	4	13.26	6.59	4–28	0.86
Positive-Difficulty identifying feelings (P-DIF)	4	10.59	5.56	4–25	0.83
Negative-Difficulty describing feelings (N-DDF)	4	14.41	6.79	4–28	0.86
Positive-Difficulty describing feelings (P-DDF)	4	11.24	5.78	4–28	0.84
General-Externally orientated thinking (G-EOT)	8	21.94	11.77	8–56	0.92
**Composites**					
General-Difficulty identifying feelings (G-DIF)^a^	8	23.85	10.63	8–52	0.87
General-Difficulty describing feelings (G-DDF)^a^	8	25.65	10.94	8–54	0.87
Negative-Difficulty appraising feelings (N-DAF)^a^	8	27.67	12.63	8–56	0.92
Positive-Difficulty appraising feelings (P-DAF)^a^	8	21.83	10.71	8–51	0.90
General-Difficulty appraising feelings (G-DAF)^a^	16	49.50	20.51	16–105	0.93
Total scale	24	71.44	29.54	24–156	0.94

*N = 370*.

a *The G-DIF and G-DDF composites are composed of a combination of the negative and positive subscales for that component. The DAF composites are composed of a combination of the DIF and DDF subscales, because theoretically the DIF and DDF components of alexithymia are closely linked [both correspond to deficits at the appraisal stage of emotion processing; (1)]*.

Our results also supported the concurrent and discriminant validity of the PAQ. In line with theoretical expectations (49), alexithymia was associated with an emotional reactivity profile comprised of high negative reactivity and low positive reactivity. High PAQ total scores were significantly associated (*p* < 0.01) with more easily activated (*r* = 0.30), more intense (*r* = 0.20) and more persistent (*r* = 0.35) negative emotions, and less easily activated (*r* = −0.16), less intense (*r* = −0.18) and less persistent (*r* = −0.23) positive emotions. The full correlation matrix is provided in Supplementary Table 2.

The PAQ and PERS were, moreover, measuring separate constructs statistically. Our second-order EFA of the PAQ and PERS subscales extracted three correlated factors (general alexithymia, positive reactivity, negative reactivity), with all the PAQ subscales loading cleanly on the general alexithymia factor (loadings = 0.70–0.81) and not loading on either of the emotional reactivity factors (loadings = −0.19–0.09; see Supplementary Table 3). As such, whilst previous work has highlighted that TAS-20 scores may be confounded by respondents' current levels of negative affect [e.g., (50)], this issue was not present for the PAQ in our data-set [see also (34, 37)].

### Conclusions, Implications, and Limitations

Overall, our results suggest that the PAQ has strong psychometric properties and can provide a robust alexithymia profile at the subscale and composite score level. Our Spanish language version appears to function similarly to the original English version in this respect, and so may be helpful in enabling cross-cultural studies [e.g., (30)] and more detailed assessments of alexithymia in Spanish-speaking populations. In psychiatric contexts, high PAQ scores could indicate cases where treatment approaches need to account for alexithymic deficits, or directly target alexithymia as part of the treatment approach [for a discussion of alexithymia treatment approaches, see (1, 41)]. A limitation of our study, however, is that our sample was from the community, so we cannot comment on psychometric performance in specialized psychiatric populations. Previous studies have found the alexithymia construct to manifest similarly across community and psychiatric samples [e.g., (17)], but it will be important for future work to test the generalizability of our PAQ findings. Similarly, future work would be beneficial to examine the test-retest reliability of the PAQ, and its concurrent validity against observer-rated, behavioral, and lab-based markers of emotion processing and other emotional constructs [e.g., (3)].

## Data Availability Statement

The raw data supporting the conclusions of this article will be made available by the authors, without undue reservation.

## Ethics Statement

The studies involving human participants were reviewed and approved by Research Committee, University of Santiago, Chile. The patients/participants provided their written informed consent to participate in this study.

## Author Contributions

RB translated the questionnaire. RB and DP contributed the majority of the manuscript writing. DP completed the statistical analyses. AF and CB recruited and administered the questionnaire to the sample, and helped refine the manuscript and translation. All authors have approved the final article and agreed to the authorship order.

## Conflict of Interest

The authors declare that the research was conducted in the absence of any commercial or financial relationships that could be construed as a potential conflict of interest.

## Publisher's Note

All claims expressed in this article are solely those of the authors and do not necessarily represent those of their affiliated organizations, or those of the publisher, the editors and the reviewers. Any product that may be evaluated in this article, or claim that may be made by its manufacturer, is not guaranteed or endorsed by the publisher.

## Footnotes

^1^Bifactor models are a theoretically congruent representation for measures that “primarily reflect a strong common trait [i.e., alexithymia], but there is multidimensionality caused by well-defined clusters of items from diverse subdomains” [(40), p. 692]. This is the case for alexithymia, which authors conceptualize as a multidimensional construct with several subcomponents (1, 40, 41).

^2^Some (*n* = 36) of the 370 participants had a small amount of missing data (i.e., data for 20% or less of the items were missing) for at least one of the administered questionnaires. In these cases, missing data were replaced using the expectation maximization method.

^3^CFA was used because it is appropriate for hypothesis testing when there is a clear theoretical rationale to expect a certain factor structure (44). In the interest of comparability with previous research, the maximum likelihood estimation method was used, as it is the most commonly used estimation method in the alexithymia field [e.g., (17, 27)], and was used in the original CFA studies of the PAQ (34). All PAQ items were reasonably normally distributed in this data-set (average skew = 0.67, average kurtosis = −0.61), so we judged the maximum likelihood method to be appropriate (45).
