# Supplementary material for: Assessing Alexithymia: Psychometric Properties of the Perth Alexithymia Questionnaire in a Spanish-Speaking Sample
Source: Front Psychiatry. 2021 Oct 12;12:710398. doi: 10.3389/fpsyt.2021.710398 (PMC8545878; doi:10.3389/fpsyt.2021.710398)
Supplement: Supplementary file 1 [file Table_1.DOCX]

**Supplementary Materials**

Table S1

*Factor Intercorrelations from Confirmatory Factor Analyses of the Perth Alexithymia Questionnaire*

|  | Factor | | | | |
| --- | --- | --- | --- | --- | --- |
|  | F1 | F2 | F3 | F4 | F5 |
|  |  |  |  |  |  |
| Five-factor model |  |  |  |  |  |
| F1 “N-DIF” | - | - | - | - | - |
| F2 “P-DIF” | .59** | - | - | - | - |
| F3 “N-DDF” | .91** | .47** | - | - | - |
| F4 “P-DDF” | .55** | .91** | .60** | - | - |
| F5 “G-EOT” | .58** | .60** | .60** | .63** | - |
|  |  |  |  |  |  |
| Bifactor model |  |  |  |  |  |
| F1 “N-DIF” | - | - | - | - | - |
| F2 “P-DIF” | .20** | - | - | - | - |
| F3 “N-DDF” | .84** | - | - | - | - |
| F4 “P-DDF” | - | .81** | .11* | - | - |
| F5 “G-EOT” | - | - | - | - | - |
| F6 “general” | - | - | - | - | - |
|  |  |  |  |  |  |

Note. ***p*<.001, **p*<.05.

Table S2
*Pearson Correlations between the Perth Alexithymia Questionnaire (PAQ) and Perth Emotional Reactivity Scale (PERS)*

|  | PERS | | | | | | | | |
| --- | --- | --- | --- | --- | --- | --- | --- | --- | --- |
|  | Subscales | | | | | |  | Composites | |
|  | Negative-Activation | Negative-Intensity | Negative-Duration | Positive-Activation | Positive-Intensity | Positive-Duration |  | Negative Reactivity | Positive Reactivity |
| PAQ |  |  |  |  |  |  |  |  |  |
| Subscales |  |  |  |  |  |  |  |  |  |
| N-DIF | .371^**^ | .289^**^ | .398^**^ | -.033 | -.095 | -.163^**^ |  | .388^**^ | -.111^*^ |
| P-DIF | .179^**^ | .093 | .232^**^ | -.144^**^ | -.101 | -.187^**^ |  | .186^**^ | -.161^**^ |
| N-DDF | .333^**^ | .253^**^ | .353^**^ | -.040 | -.099 | -.129^*^ |  | .344^**^ | -.102 |
| P-DDF | .206^**^ | .100 | .250^**^ | -.190^**^ | -.168^**^ | -.168^**^ |  | .206^**^ | -.197^**^ |
| G-EOT | .173^**^ | .108^*^ | .219^**^ | -.190^**^ | -.209^**^ | -.245^**^ |  | .184^**^ | -.242^**^ |
| Composites |  |  |  |  |  |  |  |  |  |
| G-DIF | .323^**^ | .228^**^ | .368^**^ | -.096 | -.112^*^ | -.199^**^ |  | .338^**^ | -.153^**^ |
| G-DDF | .316^**^ | .210^**^ | .351^**^ | -.125^*^ | -.150^**^ | -.169^**^ |  | .322^**^ | -.167^**^ |
| N-DAF | .372^**^ | .286^**^ | .397^**^ | -.039 | -.103^*^ | -.154^**^ |  | .387^**^ | -.113^*^ |
| P-DAF | .204^**^ | .102^*^ | .255^**^ | -.177^**^ | -.143^**^ | -.188^**^ |  | .208^**^ | -.190^**^ |
| G-DAF | .336^**^ | .230^**^ | .378^**^ | -.116^*^ | -.138^**^ | -.193^**^ |  | .347^**^ | -.168^**^ |
| Total scale | .302^**^ | .203^**^ | .350^**^ | -.156^**^ | -.179^**^ | -.232^**^ |  | .314^**^ | -.213^**^ |

*Note.* ***p*<.01, **p*<.05.

Table S3
*Factor Loadings from a Second-Order Exploratory Factor Analysis of the Perth Alexithymia Questionnaire (PAQ) and Perth Emotional Reactivity Scale (PERS) Subscale Scores to Examine Discriminant Validity*

| **Measure/ subscale** | **Factor 1 “general alexithymia”** | **Factor 2 “positive reactivity”** | **Factor 3 “negative reactivity”** |
| --- | --- | --- | --- |
| **PAQ** |  |  |  |
| N-DIF | **.726** | .081 | -.192 |
| P-DIF | **.784** | -.009 | .088 |
| N-DDF | **.720** | .080 | -.150 |
| P-DDF | **.809** | -.042 | .086 |
| G-EOT | **.699** | -.098 | .046 |
| **PERS** |  |  |  |
| Negative-Activation | .023 | -.080 | **-.870** |
| Negative-Intensity | -.066 | .046 | **-.888** |
| Negative-Duration | .099 | -.039 | **-.820** |
| Positive-Activation | -.017 | **.847** | -.116 |
| Positive-Intensity | .015 | **.876** | .016 |
| Positive-Duration | -.015 | **.767** | .164 |
|  |  |  |  |
| **Eigenvalues** | 4.13 | 2.42 | 1.92 |
| **% variance** | 37.56 | 20.38 | 17.45 |

*Note.* Principal axis factoring with direct oblimin rotation was used. Factor loadings > .40 are in boldface.
