# Supplementary material for: Assessing Alexithymia: Psychometric Properties of the Perth Alexithymia Questionnaire in a Spanish-Speaking Sample
Source: Front Psychiatry. 2021 Oct 12;12:710398. doi: 10.3389/fpsyt.2021.710398 (PMC8545878; doi:10.3389/fpsyt.2021.710398)
Supplement: Supplementary file 2 [file Data_Sheet_1.PDF]

PAQ

Nombre: **Fecha:**

Este cuestionario pregunta acerca de cómo percibes y sientes tus emociones. **Por favor indica qué tan de acuerdo o en desacuerdo estás con las siguientes afirmaciones.**

Algunas afirmaciones mencionan emociones negativas o desagradables, por ejemplo tristeza, enfado, miedo. Algunas afirmaciones mencionan emociones positivas o agradables, por ejemplo, felicidad, diversión, alegría.

		**Totalmente en desacuerdo**	**----**	**----**	**Ni de acuerdo ni en desacuerdo**	**----**	**----**	**Totalmente de acuerdo**
1	Cuando me siento mal (con una emoción desagradable), no puedo encontrar las palabras adecuadas para describir esas emociones.	1	2	3	4	5	6	7
2	Cuando me siento mal, no sé si estoy triste, enojado, o asustado	1	2	3	4	5	6	7
3	Tiendo a ignorar como me siento	1	2	3	4	5	6	7
4	Cuando me siento bien (con una emoción agradable), no puedo encontrar las palabras adecuadas para describir esas emociones.	1	2	3	4	5	6	7
5	Cuando me siento bien, no sé si estoy feliz, emocionado, o divertido.	1	2	3	4	5	6	7
6	Prefiero dejar que mis sentimientos ocurran sin prestarles atención	1	2	3	4	5	6	7
7	Cuando me siento mal, no puedo hablar acerca de esos sentimientos con profundidad o detalle.	1	2	3	4	5	6	7
8	Cuando me siento mal, no logro entender esas emociones.	1	2	3	4	5	6	7
9	No le presto atención a mis emociones.	1	2	3	4	5	6	7
10	Cuando me siento bien, no puedo hablar acerca de esos sentimientos con profundidad o detalle.	1	2	3	4	5	6	7
11	Cuando me siento bien, no logro entender esas emociones.	1	2	3	4	5	6	7
12	Trato de no pensar acerca de lo que estoy sintiendo.	1	2	3	4	5	6	7

		Totalmente en desacuerdo	----	----	Ni de acuerdo ni en desacuerdo	----	----	Totalmente de acuerdo
13	Cuando algo malo pasa, me cuesta poner en palabras como me siento	1	2	3	4	5	6	7
14	Cuando me siento mal, me confundo acerca de qué emoción estoy sintiendo.	1	2	3	4	5	6	7
15	Prefiero prestarle atención a cosas que puedo ver y tocar en vez de mis emociones.	1	2	3	4	5	6	7
16	Cuando algo bueno pasa, me cuesta poner en palabras cómo me siento.	1	2	3	4	5	6	7
17	Cuando me siento bien, confundo las emociones que estoy sintiendo.	1	2	3	4	5	6	7
18	Trato de no conectarme con mis emociones.	1	2	3	4	5	6	7
19	Si me siento mal, no sé qué decir cuando trato de explicar cómo me siento.	1	2	3	4	5	6	7
20	Cuando me siento mal, me confunden esas emociones	1	2	3	4	5	6	7
21	No es importante para mi saber lo que estoy sintiendo.	1	2	3	4	5	6	7
22	Si me siento bien, no sé qué decir cuando trato de explicar cómo me siento.	1	2	3	4	5	6	7
23	Cuando me siento bien, me confunden esas emociones.	1	2	3	4	5	6	7
24	Para mí, es extraño pensar en mis emociones.	1	2	3	4	5	6	7
